# The ratio between cerebral blood flow and Tmax predicts the quality of collaterals in acute ischemic stroke

**DOI:** 10.1371/journal.pone.0190811

**Published:** 2018-01-30

**Authors:** Ivana Galinovic, Elena Kochova, Ahmed Khalil, Kersten Villringer, Sophie K. Piper, Jochen B. Fiebach

**Affiliations:** 1 Center for Stroke Research Berlin (CSB), Charité-Universitätsmedizin Berlin, Berlin, Germany; 2 International Graduate Program Medical Neurosciences, Charité – University Medicine Berlin, Berlin, Germany; 3 Berlin School of Mind and Brain, Humboldt-Universitaet zu Berlin, Berlin, Germany; 4 Max Planck Institute for Human Cognitive and Brain Sciences, Leipzig, Germany; 5 Institute of Medical Biometrics and Clinical Epidemiology, Charité-Universitätsmedizin Berlin, Berlin, Germany; Fraunhofer Research Institution of Marine Biotechnology, GERMANY

## Abstract

**Background:**

In acute ischemic stroke the status of collateral circulation is a critical factor in determining outcome. We propose a less invasive alternative to digital subtraction angiography for evaluating collaterals based on dynamic-susceptibility contrast magnetic resonance imaging.

**Methods:**

Perfusion maps of Tmax and cerebral blood flow (CBF) were created for 35 patients with baseline occlusion of a major cerebral artery. Volumes of hypoperfusion were defined as having a Tmax delay of > 4 seconds (Tmax4s) and > 6 seconds (Tmax6s) and a CBF drop below 80% of healthy, contralateral tissue. For each patient a ratio between the volume of the CBF and the Tmax based perfusion deficit was calculated. Associations with collateral status and radiological outcome were assessed with the Mann-Whitney-U test, uni- and multivariable logistic regression analyses as well as area under the receiver-operator-characteristic (ROC) curve.

**Results:**

The CBF/Tmax volume ratios were significantly associated with bad collateral status in crude logistic regression analysis as well as with adjustment for NIHSS at admission and baseline infarct volume (OR = 2.5 95% CI[1.2–5.4] p = 0.020 for CBF/Tmax 4s volume ratio and OR = 1.6 95% CI[1.0–2.6] p = 0.031 for CBF/Tmax6s volume ratio). Moreover, the ratios were significantly correlated to final infarct size (Spearman’s rho = 0.711 and 0.619, respectively for the CBF/Tmax4s volume ratio and CBF/Tmax6s volume ration, all p<0.001). The ratios also had a high area under the ROC curve of 0.93 95%CI[0.86–1.00]) and 0.90 95%CI[0.80–1.00]respectively for predicting poor radiological outcome.

**Conclusions:**

In the setting of acute ischemic stroke the CBF/Tmax volume ratio can be used to differentiate between good and insufficient collateral circulation without the need for invasive procedures like conventional angiography.

## Introduction

In the setting of acute ischemic stroke caused by an occlusion of a cerebral artery collateral circulation is a critical factor in determining infarct evolution[1] and influencing patients’ eligibility for endovascular treatment as it has the capacity to salvage penumbral tissue through redistribution of blood via leptomeningeal anastomoses. It is known that collateral status widely differs between patients; with a number of environmental and genetic factors having been implicated in its determination[2]. The current gold standard for evaluating collateral status is digital subtraction angiography (DSA), a time-consuming and invasive method performed only in patients eligible for thrombectomy. Multiphasic CTA enables a rough classification of collaterals but requires iodinated contrast agent and x-ray use[3]. Dynamic-susceptibility contrast magnetic resonance imaging (DSC MRI) is a near non-invasive method feasible in a large percentage of the population. Some attempts have already been made to use it in categorizing collateral status[4,5]. These have relied solely on the Tmax parameter which is heavily influenced by delay and dispersion[6] and therefore combining it with additional parameters[7], such as the delay-insensitive cerebral blood flow (CBF) might be advantageous. We hypothesized that a mismatch in the volume of hypoperfused tissue between the CBF map and the Tmax map (CBF/Tmax volume ratio) will reflect the quality of collaterals, i.e. that a smaller volume of perfusion deficit on the CBF maps as compared to the Tmax map (CBF<Tmax) would be associated with good collaterals whereas a similar perfusion deficit volume on both maps (CBF≈Tmax) would be indicative of poor collaterals.

## Materials and methods

Thirty-five patients were retrospectively included from the prospective, single-center observational study 1000Plus (clinicaltrials.org NCT00715533) which received approval from the local ethics committee (Charité Ethikkommission, Ethikausschuss 4 am Campus Benjamin Franklin, Charitéplatz 1, 10117 Berlin; Institutional Review Board number EA4/026/08) and the prospective, single-center observational LOBI-BBB study (clinicaltrials.org NCT02077582, ethics approval from Charité Ethikkommission, Ethikausschuss 4 am Campus Benjamin Franklin, Charitéplatz 1, 10117 Berlin; Institutional Review Board number EA1/200/13). All patients gave written informed consent. The imaging protocol consisted of a T2*-weighted sequence, diffusion-weighted imaging (DWI), time-of-flight magnetic resonance angiography (TOF MRA), a fluid-attenuated-inversion-recovery sequence (FLAIR) and dynamic susceptibility contrast (DSC) MRI. DSC T2*-weighted images were collected using a single-shot gradient-echo EPI sequence (TE = 29ms; TR = 1390ms; pixel size = 1.8 x 1.8mm^2^; slice thickness = 5mm; interslice gap = 0.5mm) on a clinical 3 Tesla MR scanner (Tim Trio, Siemens AG, Erlangen, Germany) with a fixed dose of 5 ml of Gadobutrol (Gadovist^®^ 1 mmol/ml, Bayer Schering Pharma). Inclusion criteria were acute ischemic stroke within 24h of onset, DSC MRI at baseline and occlusion of the middle or posterior cerebral artery with no subsequent recanalization on day 2. Both baseline occlusion and subsequent lack of recanalization were assessed based on TOF MRA images. Eighteen patients had an occlusion of the middle cerebral artery (MCA; either M1 or M2 segment), 10 patients suffered a combined occlusion of the internal carotid artery and the ipsilateral MCA whereas 7 patients had an occlusion of the posterior cerebral artery (PCA). The collateral status was judged as good or poor based on the baseline Higashida score[1, 8–9], a catheter angiography rating system for assessing collateralization applied to subtracted DSC MRI. Higashida score was judged by two raters (IG and KV) with an overall agreement rate of 74% (9 discrepant cases out of 35 subjects) and inter-rater free marginal kappa of 0.66. A consensus between raters regarding the final Higashida score was reached for the 9 discrepant cases. The collateral status was considered poor if the Higashida score was 1 or 2 and good if it was 3 or 4. No patients presented with a Higashida score of 0.

CBF and Tmax perfusion maps were created in Stroketool v. 3.1. (copyright by Digital Image Solutions) using singular value decomposition. The arterial input function was selected from M2 branches of the middle cerebral artery contralateral to the side of the acute stroke. Tissue maps of cerebrospinal fluid, grey matter (GM) and white matter (WM) were created from B0 images using SPM8 (copyright 1991,1994–2015 FIL). DSC MRI was then co-registered to the B0 images using FSL FLIRT (copyright Analysis Group, FMRIB, Oxford, UK). For both the Tmax and the CBF maps a volume of interest (VOI) was loosely outlined around the visually judged area of hypoperfusion by a reader (EK) blinded to collateral status. This VOI was separated into GM and WM compartments and a threshold applied to calculate the volume of perfusion deficit. Reliable quantification of CBF from DSC MRI is known to be problematic[10] and values are likely to depend on choice of post processing method as well as show intersubject variability. Therefore, for CBF the threshold was set to 80% of normal perfusion (for grey and white matter alike) where normal perfusion was defined as the mean CBF value of the contralateral unaffected hemisphere (separately determined for grey and white matter). This threshold was chosen based on the fact that a CBF drop below this has been correlated to hemodynamic penumbra[11]. The threshold for Tmax was set at 4 sec[12] and 6 sec[13]. A ratio between the volume of tissue with reduced cerebral blood flow (a.k.a. hypoperfused tissue as based on the CBF map) and the volume of tissue with prolonged Tmax (a.k.a. hypoperfused tissue as based on the Tmax map) was calculated for each patient and will subsequently be referred to as the CBF/Tmax volume ratio. Initial and final infarct volumes were manually delineated on baseline diffusion-weighted imaging (DWI) and fluid-attenuated inversion recovery (FLAIR) images 5 to 7 days post-stroke, respectively. In four patients 5 to 7 day follow-up scans were not available so their final infarct sizes were delineated based on day 2 FLAIR images. Final infarct volume was considered equivalent with radiological outcome and was dichotomized as follows: patients with final infarct size which was equal to or greater than 1/3 of the vascular territory corresponding to their vessel occlusion were considered to have poor radiological outcome and those with final infarct size under 1/3 of the territory supplied by their acutely occluded artery were considered to have good radiological outcome. Cerebral tissue volumes matching the vascular territory of the M1 segment of the MCA, the M2 branch of the MCA and the PCA were based on literature [14,15]. Infarct growth was defined as final infarct volume on FLAIR minus baseline infarct volume on DWI. Baseline characteristics were compared between patients with good and poor collateral status using the nonparametric Mann-Whitney-U test for quantitative data and Fisher’s exact test for categorical data. Logistic regression was used to assess the strength of the CBF/Tmax volume ratios in predicting bad collateral status according to Higashida score in univariate analysis as well as with adjustment for NIHSS at admission and initial infarct volume (which were the baseline characteristics significantly different between patients with good and bad collateral status). ROC curve analysis was used to determine the strength of the CBF/Tmax volume ratios in predicting radiological outcome. ROC curves were compared using Stata function roccomp. The method exploits the mathematical equivalence of the AUC to the Mann-Whitney U-statistic[16]. Skewed parameters (initial infarct volume) were log-transformed before entering regression models. Statistical significance was set a priori at p<0.05 without adjustment for multiple comparisons. All tests should be understood as constituting exploratory data analyses. Statistical analysis was done with SPSS (IBM Corp. Released 2015. IBM SPSS Statistics for Windows, Version 23.0. Armonk, NY: IBM Corp.) and STATA/IC (Stata Statistical Software: Release 14.1. College Station, TX: StataCorp LP.)

## Results

A total of 35 patients were included into the study (median age 76 years, range 35–89, 22 females). Thirteen patients belonged to the group with good collateral status and 22 to the group with poor collaterals. Patient demographics and group comparisons are given in Table 1.

**Table 1:** Patient demographics and results.

	All patients (n = 35)	Good collaterals (n = 13)	Poor collaterals (n = 22)	p-value
Gender, Female (%)	22 (63)	10 (77)	12 (55)	0.282
Age, Years	76 (67–82)	76 (71–83)	76 (67–83)	0.880
Time from stroke to MRI (hours)	5.5 (1.4–14.9)	3.0 (1.0–10.5)	10.0 (1.9–16.1)	0.212
NIHSS admission	11.0 (4.0–14.0)	5 (1.5–11.5)	13.0 (7.0–14.3)	0.028
NIHSS discharge	11.0 (2.0–14.0)	4.0 (0.5–9.5)	12.5 (6.8–17.5)	0.002
Initial infarct size (ml)	8.7 (3.8–28.5)	1.4 (0.5–6.3)	24.3 (8.4–44.8)	<0.001
Final infarct size (ml)	34.3 (9.1–96.4)	8.3 (3.1–20.5)	73.8 (34.6–144.3)	<0.001
Infarct growth (ml)	24.6 (6.1–64.1)	2.9 (1.7–12.2)	49.5 (16.9–113.3)	<0.001
Tmax4s volume (ml)	119.7 (75.8–198.3)	119.7 (59.3–154.8)	126.3 (76.8–200.4)	0.649
Tmax6s volume (ml)	85.0 (59.8–166.1)	81.0 (46.5–109.4)	100.6 (61.2–169.4)	0.489
CBF volume (ml)	81.4 (49.7–142.4)	59.2 (27.9–75.1)	109.5 (69.5–179.3)	0.001
CBF/Tmax4s volume ratio	0.71 (0.45–1.01)	0.44 (0.36–0.60)	0.96 (0.70–1.06)	<0.001
CBF/Tmax6s volume ratio	1.00 (0.64–1.28)	0.63 (0.50–0.81)	1.16 (0.96–1.40)	<0.001

All values are medians (with limits of interquartile range). Comparisons between groups were performed using the Mann-Whitney-U test for all variables except gender for which Fisher’s exact test was used.

**Fig 1:**
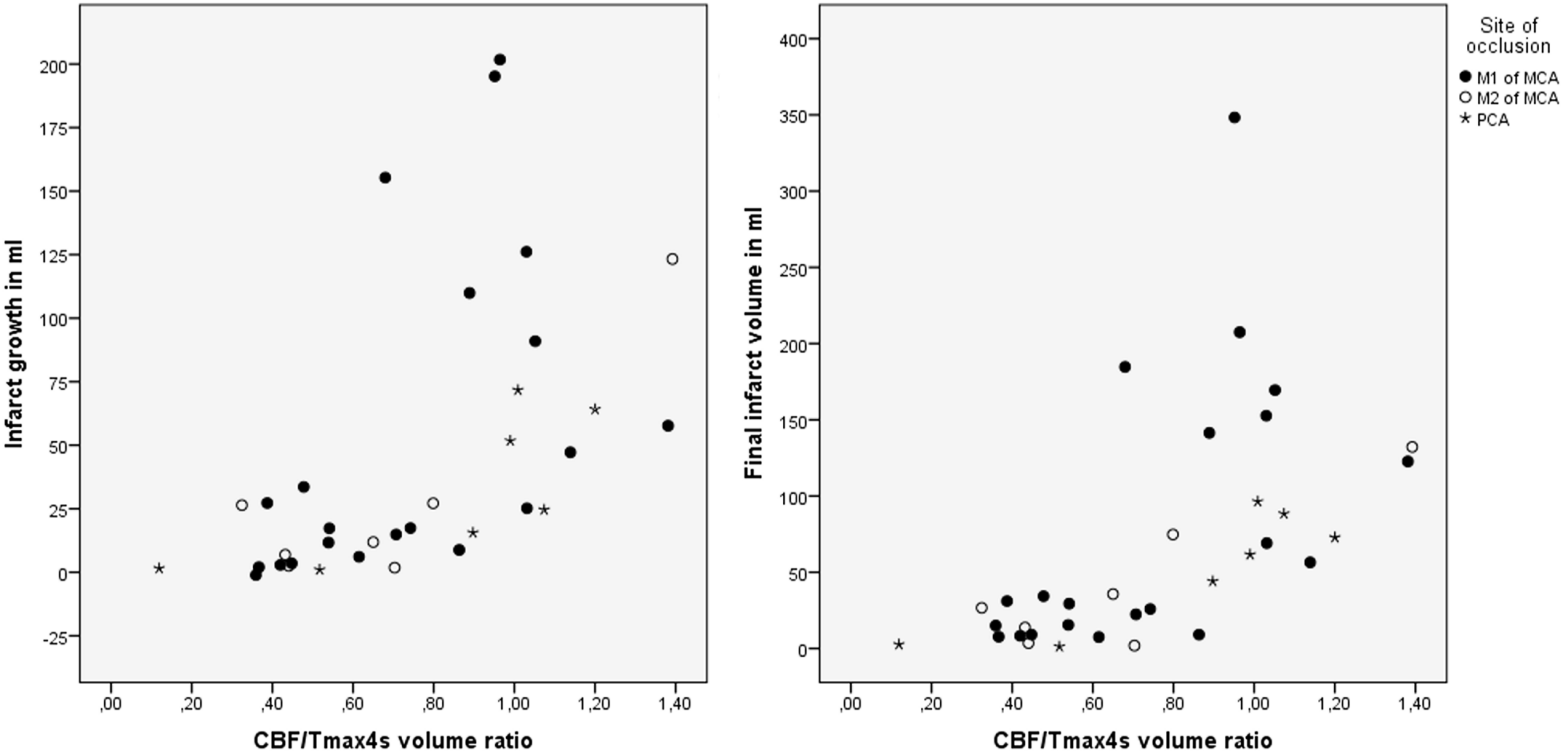
Scatter plot of the relationship between CBF/Tmax4s volume ratio, infarct growth and final infarct size, additionally divided based on type of vessel occlusion.

In our study, both Tmax thresholds produced CBF/Tmax volume ratios which performed similarly well on all accounts. The CBF/Tmax4s volume ratio was significantly associated with collateral status (Spearman’s rho = 0.720, p< 0.001) as well as correlated to infarct growth (Spearman’s rho = 0.678, p< 0.001) and final infarct volume (Spearman’s rho = 0.711, p< 0.001), for a graphic representation please see Fig 1.

The CBF/Tmax6s ratio showed similar yet slightly weaker results (Spearman’s rho = 0.650, p < 0.001 for the association with collateral status, Spearman’s rho = 0.558, p < 0.001 for the correlation with infarct growth and Spearman’s rho = 0.619, p < 0.001 for the correlation with final infarct volume).

There was a significant association between CBF/Tmax4s volume ratio and bad collateral status in the logistic regression model (OR = 2.6 95% CI[1.4–4.8] p = 0.003, pseudo R^2^ = 0.51 Accuracy 82.8% AUC 0.93 [0.85–1.00]) as well as between initial infarct volume and bad collateral status (OR = 2.6 95% CI[1.2–5.6] p = 0.014, accuracy 94.3% AUC 0.95[0.89–1.00]). CBF/Tmax4s volume ratio remained significantly associated with bad collateral status when adjusted for initial infarct size and NIHSS at admission (OR = 2.5 95% CI[1.2–5.4] p = 0.020 pseudo R^2^ = 0.65, accuracy 94.3% AUC 0.96 [0.90 1.00]) for every 0.1 increase in the CBF/Tmax4s volume ration. Analysis of the CBF/Tmax6s volume ratio gave similar results although with a somewhat weaker association to bad collateral status (OR = 1.7 95% CI[1.2–2.5] p = 0.003, pseudo R^2^ = 0.38, accuracy 82.9% AUC 0.89 [0.78–0.99]). The CBF/Tmax6s volume ratio also remained significantly associated with bad collateral status when adjusted for initial infarct size and NIHSS at admission (OR = 1.6 95% CI[1.0–2.6] p = 0.031 pseudo R^2^ = 0.58, accuracy 91.4% AUC 0.96[0.90–1.00] for every 0.1 increase in the CBF/Tmax6s volume ratio.

The CBF/Tmax volume ratios were significantly higher for patients with bad radiological outcome (median with IQR for CBF/Tmax4s volume ratio and CBF/Tmax6s volume ratio, respectively: 0.52 [0.40–0.71] and 0.72 [0.53–1.02] for patients with a good radiological outcome and 1.00 [0.90–1.11] and 1.24 [1.05–1.40] for patients with a bad radiological outcome, all Mann Whitney U tests p < 0.001). Of all the baseline parameters only initial infarct size was also significantly higher in subjects with bad radiological outcome (median with IQR: 29.0 [9.6–64.1]) as compared to good radiological outcome (5.4 [1.0–8.5]), Mann Whitney U test p < 0.001. In a logistic regression model with adjustment for initial infarct size the CBF/Tmax4s volume ratio remained significantly associated with poor radiological outcome (OR = 1.9 95% CI[1.1–3.3] p = 0.021 R^2^ = 0.62 but the CBF/Tmax6s volume ratio did not (OR 1.2 95% CI[0.9–1.5] p = 0.222 R^2^ = 0.46).

**Fig 2:**
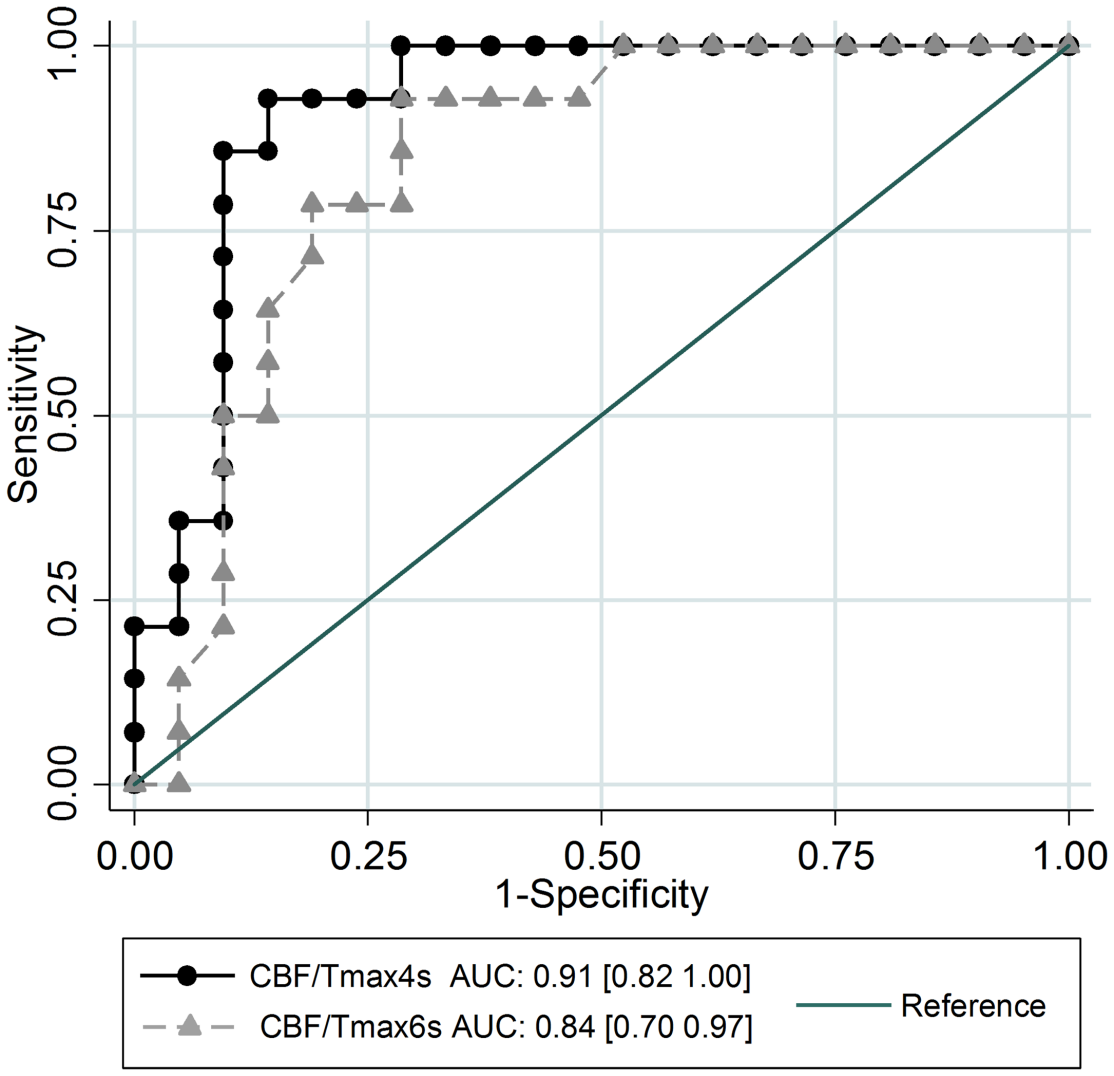
Receiver operating characteristic (ROC) curves shown for the two CBF/Tmax volume ratios illustrating the performance of various cut-offs to identify patients with poor radiological outcome.

Fig 2 shows the ROC curve for the CBF/Tmax4s volume ratio (AUC = 0.91 95% CI[0.82–1.00]) and the CBF/Tmax6s volume ratio (AUC = 0.84 95% CI[0.70–0.97]) in predicting poor radiological outcome.

In the ROC analysis the cut-off with the best accuracy for the CBF/Tmax4s volume ratio and the CBF/Tmax6s volume ratio in predicting poor radiological outcome was 0.398 (sensitivity 92.9%, specificity 85.7%) and 0.372 (sensitivity 92.9%, specificity 71.4%), respectively (Fig 2).

## Discussion

**Fig 3:**
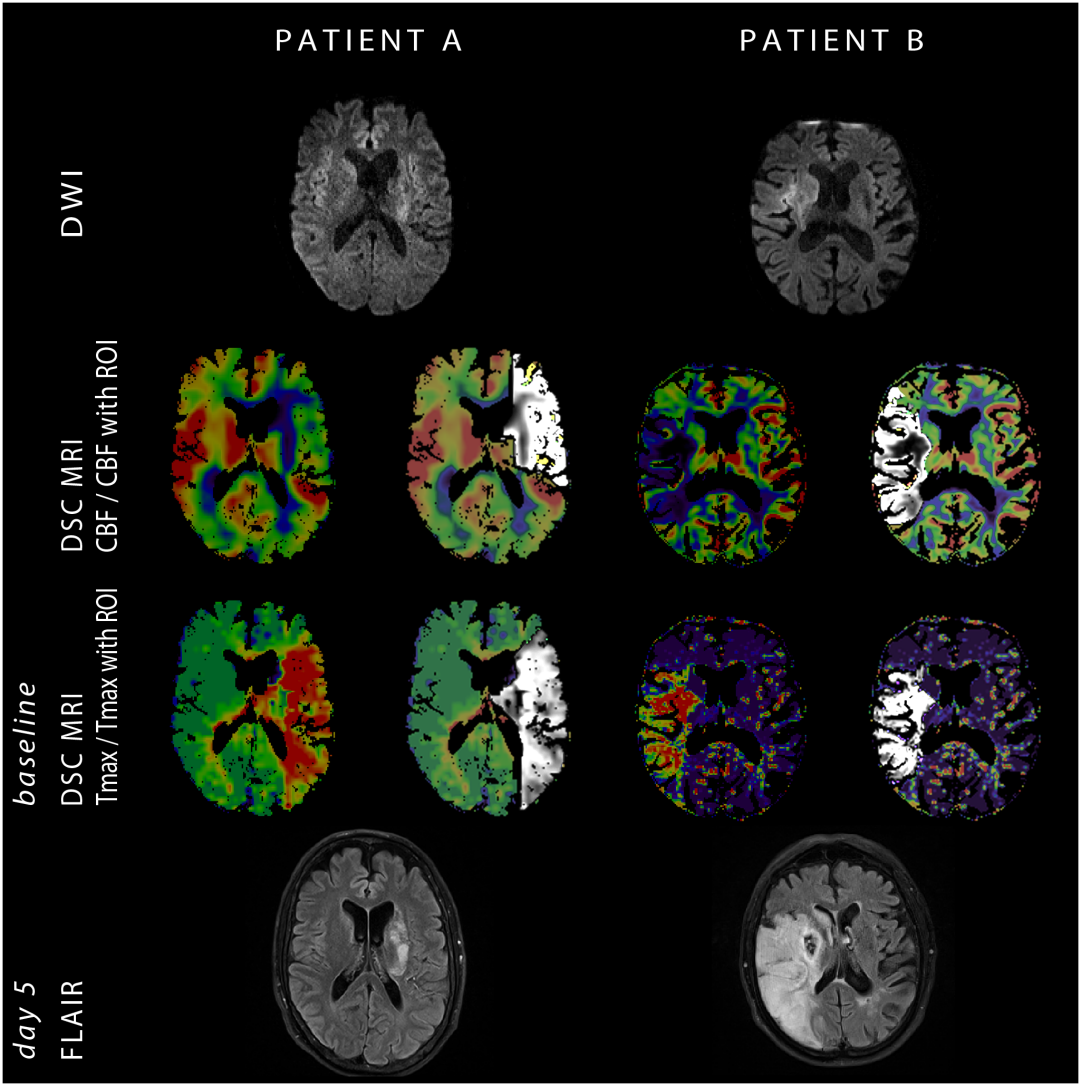
Comparison of two patients both imaged 1 hour after symptom onset with a proximal occlusion of the middle cerebral artery and a Higashida score of 2 on source DSC MRI. Patient A had a left-sided stroke with NIHSS at admission of 12, an initial infarct volume of 3.8ml, perfusion deficit of 200ml on Tmax4s and 108ml on CBF with a CBF/Tmax4s volume ratio of 0.54. The final infarct size was 15.5ml. Patient B had a right-sided stroke with NIHSS at admission of 19, an initial infarct volume of 5.8ml, perfusion deficit of 87ml on Tmax4s and 84ml on CBF with a CBF/Tmax4s volume ratio of 0.96. The final infarct size was 208ml.

This study has shown that, in the setting of acute ischemic stroke, the relationship between a perfusion deficit on a Tmax and a CBF map is associated with an individual’s collateral status. While Tmax showed comparable hypoperfusion volumes in patients with good and poor collateral circulation, CBF differentiated between them by showing a near-match in patients with insufficient collaterals and a clear mismatch in individuals with substantial collateral circulation. The authors are aware of several limitations of the study, the first being its small sample size. Another limitation was the absence of digital subtraction angiography information in our cohort, making it necessary to use a surrogate parameter in order to classify patients’ collateral status. For this purpose we chose Higashida score applied to subtracted DSC MRI, a method which has been previously published as showing good correlation to DSA [9]. Having been aware of the limitations of this method we also performed an analysis correlating our perfusion volume ratio to dichotomized radiological outcome based on final infarct size. This analysis showed all the same that a relationship between a perfusion deficit on a Tmax and a CBF map is indicative of a patient’s outcome. Another possible limitation of our study was the choice of specific CBF and Tmax thresholds. Despite extensive research into the topic, thus far no one threshold has been identified as optimal[17] for defining hypoperfusion but several are considered credible and therefore frequently used. In this study a uniform cut-off value for all subjects was necessary in order to avoid (unintentional) bias when calculating volumes of hypoperfused tissue. The particular thresholds chosen correspond to those previously used in literature. Specifically we have investigated two different thresholds for defining hypoperfused tissue on the Tmax map (4s and 6s), and have achieved comparable results using both. It was not the scope or the purpose of this study to separately evaluate all reasonable cut-offs and their possible combinations but rather to verify an observed pattern of behavior between the CBF and the Tmax map. In our population initial infarct size was also a good predictor of collateral status irrespective of the time window; a finding which is logical and has been previously reported[8]. Therefore one could argue that the appearance of the stroke on the baseline DWI could, in itself, be a sufficient clue as to the underlying functionality of collateral circulation. However, ischemia is a dynamic process and, particularly in the early time window, the still-developing lesion on DWI can be misleadingly small (Fig 3). Especially in such patients the DSC MRI based method proposed in this article can offer easily accessible and decisive information even when clinical parameters, initial appearance on DWI and other surrogate markers for evaluating collateral circulation prove unable to classify patients (Fig 3).
